# Florid reactive periostitis of the clavicle: A case report and literature review

**DOI:** 10.1097/MD.0000000000036674

**Published:** 2023-12-15

**Authors:** Jinshuo Tang, Xuemei Wang, Enbo Liu, Zhixin Niu, Jianlin Zuo, Tong Liu, Hongwei Li

**Affiliations:** a Department of Orthopaedics, China-Japan Union Hospital of Jilin University, Changchun, Jilin, China; b Department of Pathology, China-Japan Union Hospital of Jilin University, Changchun, Jilin, China; c Department of Orthopaedics, Jiaozuo People’s Hospital, Jiaozuo, Henan, China.

**Keywords:** clavicle, diagnosis, florid reactive periostitis, FRP, treatment

## Abstract

**Rationale:**

Florid reactive periostitis (FRP), a rare reactive bone lesion, typically presents in the short tubular bones of the extremities, with infrequent occurrences in the long tubular bones. This report discusses a unique case of FRP in the clavicle, managed through comprehensive lesion debridement and bone grafting, yielding positive results over a 3-year duration.

**Patient concern:**

A 25-year-old male presented with a discernible mass at the left sternal end of the clavicle, discovered incidentally 2 weeks prior. The patient exhibited no clinical signs of inflammation, pain, sinus tract, or suppuration.

**Diagnosis:**

Initial pathological examination of the local excision suggested benign lesions, although malignancy could not be ruled out. A definitive diagnosis of clavicular FRP was reached post complete lesion resection, with supporting evidence from postoperative pathology, imaging, and clinical symptoms.

**Intervention:**

The left clavicle was reconstructed through an open surgical procedure involving total mass removal and ipsilateral extraction of an iliac bone of suitable dimensions. This was implanted into the clavicular bone defect and internally fixed with a plate.

**Outcomes:**

Three years of consecutive follow-up revealed no recurrence of hyperplasia, absence of mass or tenderness at the left sternal end of the clavicle, and unimpaired function of adjacent joints.

**Lessons:**

The primary clinical challenge with FRP is its diagnosis. While pathological diagnosis remains crucial, it is also important to incorporate imaging and clinical symptoms for a comprehensive assessment. Complete mass excision may offer specific benefits in distinguishing FRP from its malignant counterparts.

## 1. Introduction

A plethora of fibrous osseous and osteochondral anomalies, typically identified within the short tubular bones of the hands and feet, present a diagnostic conundrum and have traditionally been classified as reactive lesions.^[1]^ Florid reactive periostitis (FRP), a rare type of reactive lesion found in bone, primarily manifests in the short tubular bones of the extremities. There have been sporadic case reports of FRP linked with long tubular bones.^[2]^ The typical age of onset for FRP is between 20 and 40 years, although literature reports a broader age range from 5 to 70 years. The condition appears to be more prevalent among adolescents and young adults, with a slight predominance in females compared to males.^[3]^ Clinical manifestations of FRP encompass localized discomfort and soft tissue edema, occasionally accompanied by tenderness and erythema during the initial stages. Radiographic representation of this benign lesion usually exhibits soft tissue edema, occasionally coupled with soft tissue calcification, and a periosteal response is generally observed.^[4]^

Although FRP is a benign lesion, it presents diagnostic challenges, especially because its histopathological features may resemble those of malignant tumors such as osteosarcoma. Therefore, we herein report a case of FRP of long tubular bone admitted to our hospital, thus providing a reference for the clinical management of such patients.

## 2. Case description

A 25-year-old male patient was admitted to our medical facility with a palpable mass at the left sternal end of the clavicle, which he had observed 2 weeks prior, absent any clinical indicators of inflammation, pain, sinus tracts, or suppuration. The patient negated any previous trauma history. There was no tenderness elicited upon palpation of the left sternal end of the clavicle, and no significant abnormalities in skin sensation were noted.

**Figure 1.**
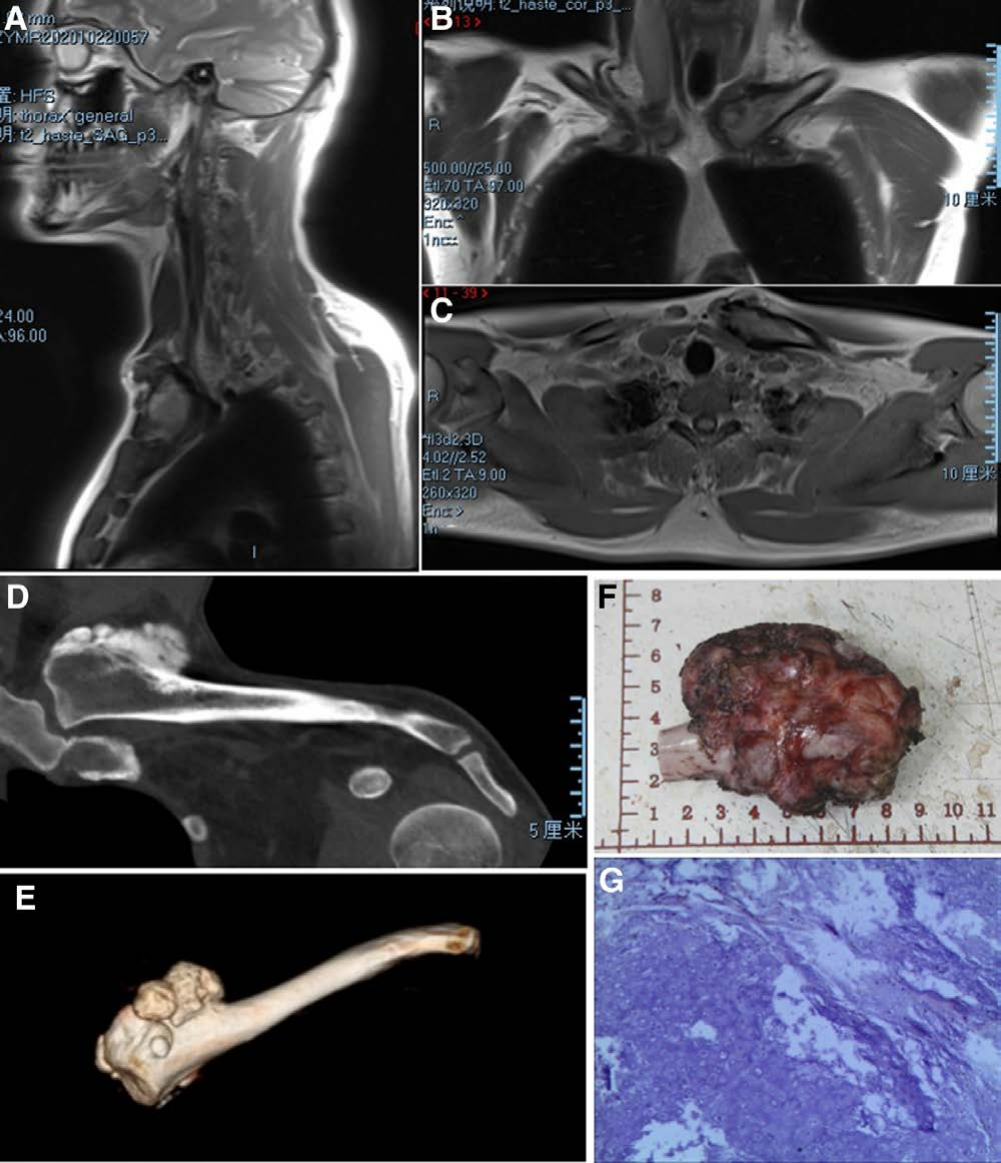
Patient’s admission MRI (A–C) and CT (D and E) imaging, gross specimen of surgically removed lesion (F), and pathology (G). CT = computed tomography, MRI = magnetic resonance imaging.

Computed tomography (CT) imaging revealed extensive osseous destruction at the left sternal end of the clavicle, with heterogeneous density within the lesion, sclerotic osteophytes at the periphery, and nodular hyperdense shadows surrounding the lesion (Fig. 1D and E). The morphology of the remaining left clavicle was essentially normal. Given these changes at the sternal end of the clavicle, our preliminary clinical diagnosis leaned towards a benign neoplasm, possibly of cartilaginous origin, prompting further investigation via magnetic resonance imaging (MRI). The MRI demonstrated an abnormal signal at the left sternal end of the clavicle, featuring expansile growth and multiple nodular lesions with localized cortical thickening, heterogeneous internal signals, low signal intensity on T2-weighted images, and localized areas of high signal intensity on fat-suppressed images, compressing the surrounding soft tissues (Fig. 1A–C). The combined CT and MRI findings suggested a benign neoplastic lesion with a likely cartilaginous origin. Meanwhile, the patient’s hematology showed no significant abnormalities.

Post obtaining informed consent from the patient, an open surgical biopsy was performed to obtain pathological insights. Intraoperatively, a substantial amount of cartilage-like osteophytes with a cauliflower-like appearance was observed at the sternal end of the clavicle. The histopathological examination of the excised specimen revealed it to be hyperplasia of braided trabeculae with occasional nuclear schizophrenia and some areas of chondrocyte mucous degeneration with mild anisotropy (Fig. 1G). However, these findings did not conclusively rule out a malignant neoplasm.

**Figure 2.**
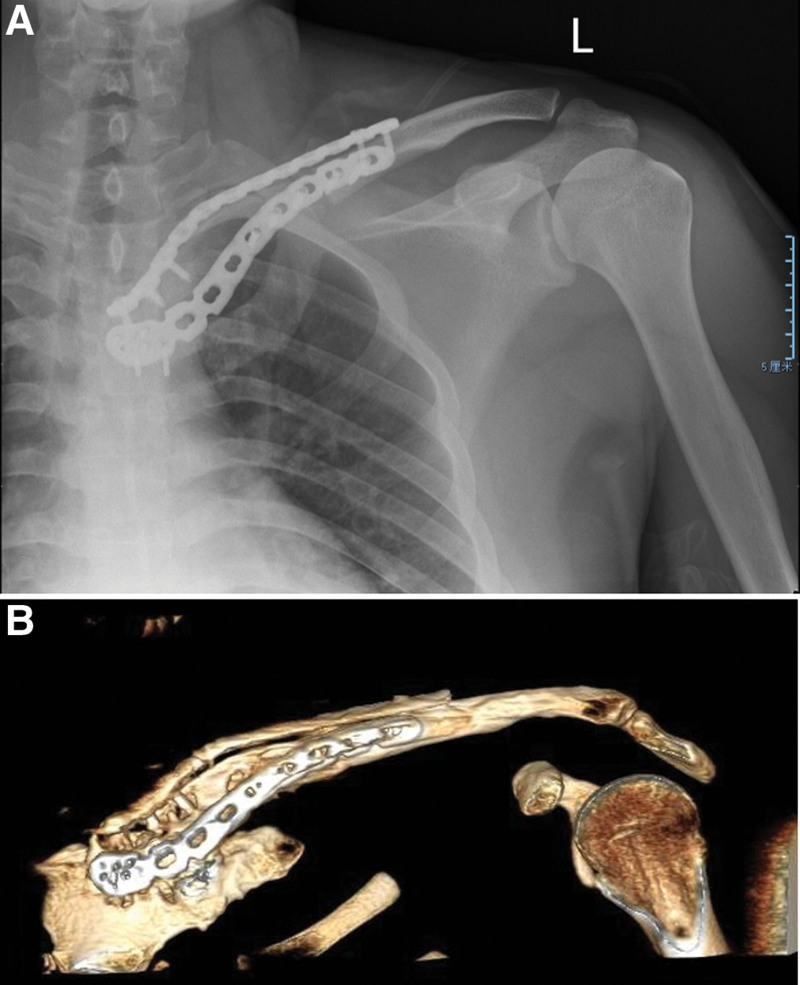
Total mass removal and ipsilateral extraction of an iliac bone of suitable dimensions. This was implanted into the clavicular bone defect and internally fixed with a locking plate (A). No recurrence of lesions during follow-up (B).

Considering the patient’s age and treatment preference, a second surgery was performed, involving a wide resection of the left sternal end of the clavicle. Intraoperatively, the proximal clavicle mass was noted to be firm with ill-defined borders with surrounding tissues and had cartilage caps attached, presenting a cauliflower-like appearance. The clavicle was resected 3 cm distal to the mass edge. The resected mass measured approximately 8 cm in length and 6 cm in width (Fig. 1F). A bone graft from the ipsilateral side ilium was used to reconstruct the clavicular defect, which was then stabilized with a plate to restore clavicle alignment (Fig. 2A).

The histopathological examination of the thoroughly resected specimen confirmed the lesion to be a mixture of spindle cell and osteoblastic tissue types. The spindle cells exhibited a myofibroblast-like morphology, and the interstitial matrix was loose and mucinous with vascular reactive hyperplasia. Cellular heterogeneity was minimal, with mature osteogenesis and nodular distribution. Spindle-shaped cells were located centrally within the nodules, with osteoblasts at the periphery, indicative of a zoning and maturation pattern. This histomorphology necessitated careful differentiation from low-grade osteosarcoma. Immunohistochemical analysis showed p53-, p63-, STAT6-, B-catenin-, and Ki-67 index < 5%. Thus, based on the pathological diagnosis and imaging findings, we diagnosed the left clavicle lesion as FRP.

Postoperative follow-up was conducted regularly. The patient’s postoperative course was favorable, with no recurrence or metastasis detected over a 3-year follow-up period (Fig. 2B). The patient demonstrated good mobility and function in the left upper extremity.

## 3. Discussion

In 1981, the term “florid reactive periostitis” was coined by Spjut HJ and Dorfman HD to describe a benign lesion that bears resemblance to osteosarcoma.^[5]^ Despite its relative rarity, the condition has been reported in the literature under various nomenclatures, including fasciitis ossificans, benign fibro-osseous pseudotumor, parosteal fasciitis, bizarre parosteal osteochondromatous proliferation, and pseudomalignant osseous tumor of soft tissue.^[6]^ The disease is most prevalent in the second and third decades of life, with a slightly higher incidence in females than males. The age range reported in literature spans from 5 to 70 years, but adolescents are more commonly affected.^[3]^

Historically, FRP was reported to predominantly affect the short tubular bones of the extremities, with infrequent involvement of long bones. However, recent studies suggest an increasing detection of FRP in long tubular bones.^[6–8]^ For instance, Jamshidi K et al diagnosed 7 cases of FRP in long bones involving the humeral shaft, proximal radius, femoral shaft, and distal femur over an 11-year period.^[4]^ Park HE et al described a case of FRP of the right clavicle in a 26-year-old male patient,^[9]^ which closely mirrors our case and represents a rarer instance of FRP of the long tubular bone occurring outside of the extremities.

Reactive bone lesions can pose diagnostic challenges as a history of prior trauma may not always be present and imaging and histopathologic findings can vary depending on the maturity of the lesion and time since injury.^[2]^ Although the definitive etiology of FRP is unknown, trauma is often considered a primary predisposing factor, with a history of trauma reported in approximately 40% of cases.^[4,10]^ However, some patients do not report a history of trauma,^[9,11]^ underscoring the importance of careful evaluation of clinical history, radiology, and pathology in reaching an accurate diagnosis. Despite its rarity, FRP should be considered in the differential diagnosis of any osteogenic growth lesion in long tubular bone. Awareness of the aforementioned differences can aid in distinguishing benign lesions from their malignant counterparts.^[12]^

Clinically, FRP occurring in long tubular bones usually presents as soft tissue swelling, occasionally with a gradual increase in pain over weeks or months. The “maturation” of the periosteal reaction can occur very rapidly due to the reactive proliferative characteristics of FRP, leading to rapid hardening of the mass within a few weeks.^[1,9]^ The clinical symptoms of the case reported by Byun BH et al differed from previous FRPs in long bones, which typically present with pain or swelling in the affected limb.^[8]^

Our comprehensive analysis indicates that FRP in long tubular bones shares similarities with FRP in short tubular bones, such as a period of acute response followed by regression of some lesions after the peak phase. Residual exostoses gradually become visible,^[4]^ and the patient’s pain symptoms subside or diminish. Hematologic tests are important in the evaluation of bacterial infectious diseases such as osteomyelitis.^[13]^ Routine laboratory tests for FRP, including serology and hematology, are typically normal,^[4]^ with normal WBC and negative CRP, as seen in our case. This aids in differentiating FRP from acute inflammatory diseases.

Imaging typically shows diffuse calcification of the lesion with periosteal reactive new bone, soft tissue edema, cortical integrity, and inseparability from mass tissue on CT. MRI reveals periosteal reaction with thickening of the bone cortex, mild enhancement of the subperiosteal area, and edema of surrounding tissues. Over time, the periosteal reaction matures, leading to FRP ossificans.^[1]^ The imaging manifestations of FRP involving long tubular bone are not significantly different from those involving short tubular bone. Skeletal X-rays show a soft tissue mass adjacent to the bone, displaying varying degrees of ossification and periosteal reaction. Cortical irregularities and erosions may raise suspicion for malignant lesions,^[9]^ although reports suggest that the cortex is usually intact with no signs of bony destruction.^[1]^ In conclusion, rapid periosteal osteogenesis without alteration of soft tissue quality may be the most typical manifestation of FRP.^[9]^ For example occurring in Jamshidi K reported FRP of long tubular bone with periosteal reaction and peri lesion edema in all cases. Calcified mass, bone marrow edema and cortical erosions were observed in 6 out of 7 patients. In addition, 2 peripheral mineralized and zoned lesions were observed.^[4]^

Histologically, the resemblance of FRP to tumor progression necessitates careful exclusion of malignancy. Differential diagnoses include parosteal osteosarcoma, low-grade osteosarcoma, chondrosarcoma, and periosteal osteosarcoma. Other conditions often considered in association with this lesion are osteomyelitis and osteochondroma.^[14]^ FRP comprises a mixture of osteoid, cartilage, bone, and fibrous tissue, and its histological features may be confused with osteomyelitis and various tumors.^[8]^ At high magnification, the lesions are characterized by reactive proliferation of spindle-shaped fibroblasts and osteoblasts with new bone formation. Cartilage lesion and large amounts of osteoid are common. The cartilage lesion is usually multicellular, consisting of an increased number of binucleated chondrocytes. However, no cytologic heterogeneity is present. The atypical mitotic figure is equally indeterminate.^[1]^ Osteoblasts may be atypical and larger in the area of osteoid production, but there is homogeneity and no interstitial lesions are found to distinguish them from osteosarcoma. As in myositis ossificans, some lesions may show a zonular phenomenon, with a central portion of osteoid surrounded by mature bone. Finally, multinucleated giant cells and inflammatory cells may be found.^[15]^ Pathologically, the histomorphology of our case was first considered as a reactive proliferative lesion such as FRP, as well as other lesions such as late stage osteomyelitis, but it was necessary to differentiate low grade osteosarcoma.

Despite the diagnostic challenges posed by many fibrous bone and osteochondral lesions, which have historically been considered reactive, modern molecular techniques supplemented with clinical, radiographic, and histologic evaluations suggest that they may indeed be neoplasms. Among similar benign lesions such as FRP, subungual exostosis, and bizarre parosteal osteochondromatous proliferation, only FRP may be a truly benign reactive bone lesion due to the absence of reproducible molecular defects.^[1]^ Despite this, the choice of therapeutic measures for FRP should be emphasized. Some studies suggest limb rest and anti-inflammatory treatment as optional measures for the treatment of FRP in long tubular bone. In all their cases, the lesions spontaneously regressed, leaving residual exostoses.^[4]^ However, given the difficulty in differentiating FRP from malignant tumors such as osteosarcoma, the typical age range of affected patients (20–40 years), and the possibility of disease recurrence,^[8]^ we recommend complete surgical resection of FRP to avoid misdiagnosis and mistreatment. In our case, based on the clinician’s judgment and the patient’s request, surgical resection was performed. After 3 years of follow-up, no recurrence was observed.

In conclusion, there are relatively few clinical reports on FRP occurring in long tubular bone. The clinical manifestations, imaging, and pathological manifestations of this disease vary across different literatures, and there are no unified and standardized clinical diagnosis and treatment guidelines and consensus for this disease. Presently, case reports of FRP in long tubular bone can inform clinical management of such diseases. In particular, the even rarer occurrence of FRP in the clavicle that we reported may be more clinically informative. We suggest that diagnosis of FRP in long tubular bones be based on pathology, and treatment options should include complete surgical resection to completely exclude the possibility of its malignant mimickers. Regular and adequate follow-up is essential to exclude recurrence and determine prognosis.

## Acknowledgments

We would like to thank ChatGPT for English language editing.

## Author contributions

**Conceptualization:** Jianlin Zuo, Tong Liu.

**Data curation:** Jinshuo Tang, Xuemei Wang, Enbo Liu, Zhixin Niu.

**Formal analysis:** Jinshuo Tang, Enbo Liu.

**Funding acquisition:** Jianlin Zuo, Tong Liu.

**Investigation:** Xuemei Wang, Zhixin Niu.

**Methodology:** Jinshuo Tang, Enbo Liu.

**Project administration:** Tong Liu.

**Resources:** Tong Liu.

**Supervision:** Jianlin Zuo, Hongwei Li.

**Software:** Jinshuo Tang, Enbo Liu.

**Validation:** Jianlin Zuo, Hongwei Li.

**Visualization:** Jinshuo Tang, Enbo Liu.

**Writing – original draft:** Jinshuo Tang, Enbo Liu.

**Writing – review & editing:** Tong Liu.

## References

[1] Memon RA Wei S Siegal GP. Some reactive lesions of bone are probably neoplasms. Arch Pathol Lab Med. 2022;146:60–9. 33946096 10.5858/arpa.2020-0817-RA

[2] Levy JL Louka KL Cooper K. Focal reactive periostitis ossificans in a long bone: radiologic and pathologic findings. Radiol Case Rep. 2021;16:3638–42. 34630790 10.1016/j.radcr.2021.09.009 PMC8495033

[3] Solana J Bosch M Español I. Florid reactive periostitis of the thumb: a case report and review of the literature. Chir Main. 2003;22:99–103. 12822245 10.1016/s1297-3203(03)00019-2

[4] Jamshidi K Givehchian B Mirzaei A. Florid reactive periostitis of the long bone: a case series of seven patients. J Orthop Sci. 2017;22:560–5. 28081927 10.1016/j.jos.2016.12.015

[5] Spjut HJ Dorfman HD. Florid reactive periostitis of the tubular bones of the hands and feet A benign lesion which may simulate osteosarcoma. Am J Surg Pathol. 1981;5:423–33. 6945056 10.1097/00000478-198107000-00002

[6] Porcel López MT Fernández Gil MA Campos de Orellana A. Florid reactive periostitis ossificans of the distal ulna. Orthopedics. 2008;31:286. 19292223 10.3928/01477447-20080301-27

[7] Brien EW Zahiri CA Mirra JM. Florid reactive periostitis ossificans of the proximal aspect of the tibia: a lesion that must be distinguished from osteosarcoma. A case report. J Bone Joint Surg Am. 1999;81:1002–7. 10428133 10.2106/00004623-199907000-00014

[8] Byun BH Koh JS Yoo JY. (99m)Tc-MDP- and (18F)-FDG-avid florid reactive periostitis ossificans mimicking recurrent osteosarcoma. Clin Nucl Med. 2013;38:482–3. 23603597 10.1097/RLU.0b013e31828da632

[9] Park HE Chai JW Jo CH. Florid reactive periostitis of the clavicle: a case report. Taehan Yongsang Uihakhoe Chi. 2022;83:414–9. 36237924 10.3348/jksr.2021.0108 PMC9514431

[10] Tomori Y Ohashi R Naito Z. Florid reactive periostitis in the fifth phalange of a professional boxer: a case report. Medicine (Baltim). 2016;95:e5697. 10.1097/MD.0000000000005697 PMC5181827 28002343

[11] Gao Z Wang J Wang Z. Florid reactive periostitis of the metacarpal and phalanx: 2 case reports. J Hand Surg Am. 2013;38:2134–7. 24206975 10.1016/j.jhsa.2013.08.115

[12] Soni A Weil A Wei S. Florid reactive periostitis ossificans of the humerus: case report and differential diagnosis of periosteal lesions of long bones. World J Orthop. 2015;6:559–63. 26301184 10.5312/wjo.v6.i7.559 PMC4539478

[13] Harris JC Caesar DH Davison C. How useful are laboratory investigations in the emergency department evaluation of possible osteomyelitis? Emerg Med Australas. 2011;23:317–30. 21668719 10.1111/j.1742-6723.2011.01413.x

[14] Nance KV Renner JB Brashear HR. Massive florid reactive periostitis. Pediatr Radiol. 1990;20:186–9. 2191267 10.1007/BF02012969

[15] Azorín D López-Pino MA González-Mediero I. Long bone florid reactive periostitis ossificans: a case in the distal femur mimicking osteosarcoma. J Pediatr Orthop B. 2008;17:301–5. 18841064 10.1097/BPB.0b013e328311d4b9

